# Personal

**Published:** 1906-05

**Authors:** 


### PERSONAL.

Dr. William M. Bemus, of Jamestown, lately appointed by the Mayor a member of the board of health of that city, was elected president of the board at its last meeting.

---

Dr. L. S. Higgins, Jr., assistant physician at the Manhattan State Hospital, New York, has been appointed assistant surgeon of the New Yohk State Soldiers' and Sailors' Home at Bath, vice Dr. Laurence J. Gerold, deceased.

---

Dr. H. M. Edmunds of Tonawanda, N. Y., has been elected by the board of health to the office of health physician of that city.

---

Dr. A. J. Martin, of North Tonawanda, N. Y., a graduate of the University of Buffalo, 1887, has been elected by the board of health to the office of health physician of that city.

---

Dr. Grosvenor R. Trowbridge, of Buffalo, announces the removal of his residence and office from 1349 Main Street to 410 Elmwood Avenue. Hours: 8 to 9, 1 to 3 and 6 to 7. Telephone, Bryant 762.

---

Dr. W. B. Gifford, of Attica, N. Y., a member of the homœpathic state board of medical examiners, has suffered a serious illness of late, but as these lines are passing through the press his condition is reported to be considerably improved, with prospects of ultimate recovery.

Dr. Albert Vander Veer, of Albany, attended the International Medical Congress at Lisbon, April 19-26, 1906, at which he read a paper. He sailed in company with Mrs. Vander Veer March 3, going to Naples, Rome, and other important cities in the south of Europe, and is expected to return to America May 9, 1906.

---

Dr. James W. C. Ely, dean of the medical profession in Providence, R. I., will shortly complete sixty years in the practice of medicine. His fellow practitioners will give him a dinner to commemorate the occasion.

---

Dr. Charles G. Stockton, of Buffalo, is spending a few weeks in Europe for rest and recreation. He is touring with friends in an automibile a portion o fthe time, and expects to return in season to attend the meeting of the American Medical Association early in June.

---

Dr. F. Park Lewis, of Buffalo, sailed for Europe May 8, 1906, to be absent until September. He was accompanied by his family,—wife and three children,—and will take the Mediterranean route, afterward touring the continent and Great Britain in a leisurely way.

---

Dr. James A. Mac Leod, of Buffalo, announces that after May 1, 1906, his office will be located at 482 Delaware Avenue. He, however, will continue to reside at the Hotel Touraine. Hours: 1 to 3 P. M.; otherwise by appointment.

---

Dr. Joseph M. Mathews, of Louisville, Ky., attended the annual meeting of the Medical Society of the State of Arkansas, held at Hot Springs, May 8, 9 and 10, 1906, at which he was the guest of honor. On the evening of the ninth he delivered a popular lecture to which invitations to the citizens in general were issued.

---

Dr. Floyd S. Crego, of Buffalo, has removed his office and residence from 469 to 503 Delaware Avenue. Hours: 9 to 10, and 1 to 3.

---

Dr. Charles W. Pilgrim, Superintendent of the State Hospital at Poughkeepsie, has been appointed by Governor Higgins president of the state commission in lunacy, vice Dr. William Mabon, resigned. Dr. Pilgrim is one of the best known alienists of the county, and during his earlier years was one of the assistants of the Utica State Hospital.

Dr. J. F. Munson, a graduate of the University of Michigan, for two years an assistant to Professor Victor C. Vaughan, has been appointed resident pathologist at the Craig Colony for Epileptics, Sonyea, N. Y.

---

Dr. Marshall Clinton, of Buffalo, has removed his office and residence from 466 Franklin Street to 516 Franklin Street. His hours and telephone number remain the same as before.

---

Dr. A. L. Benedict of 156 W. Chippewa St., (Roanoke Hotel) Buffalo, announces that after May 1, his Bell Telephone number will be Tupper 1115, the same as for the Roanoke Hotel.

---

Dr. William Ward Plummer, of Buffalo, has removed from 403 Delaware Avenue to 523 Franklin Street, the latter being the residence formerly occupied by Dr. Abbott.

